# General Practitioner trainers prescribe fewer antibiotics in primary care: Evidence from France

**DOI:** 10.1371/journal.pone.0190522

**Published:** 2018-01-25

**Authors:** Louise Devillers, Jonathan Sicsic, Angelique Delbarre, Josselin Le Bel, Emilie Ferrat, Olivier Saint Lary

**Affiliations:** 1 Department of Family Medicine, Faculty of Health Sciences Simone Veil, University Versailles Saint-Quentin en Yvelines (UVSQ), Montigny-le-Bretonneux, France; 2 CESP, University Paris-Sud, UVSQ, INSERM U1018, University Paris-Saclay, Villejuif, France; 3 Department of Family Medicine, University Paris Diderot, Sorbonne Paris Cité, Paris, France; 4 UMR 1137, INSERM, IAME, Paris, France; 5 University Paris-Est Créteil (UPEC), School of Medicine, Primary Care Department, Créteil, France; 6 University Paris-Est Créteil (UPEC), DHU A-TVB, IMRB, EA 7376 CEpiA (Clinical Epidemiology And Ageing Unit), Créteil, France; University of Brescia, ITALY

## Abstract

**Purpose:**

Antibiotic prescription is a central public health issue. Overall, 90% of antibiotic prescriptions are delivered to patients in ambulatory care, and a substantial proportion of these prescriptions could be avoided. General Practitioner (GP) trainers are similar to other GPs in terms of sociodemographic and medical activities, but they may have different prescription patterns. Our aim was to compare the antibiotic prescribing rates between GP trainers and non-trainers.

**Methods:**

This observational cross-sectional study was conducted on administrative data claims from the French National Health Insurance. The antibiotic prescribing rate was calculated. The main independent variable was the training status of the GPs. Prescribing rates were adjusted for the various GPs’ characteristics (gender, age, location of the practice, number of visits per GP and the case-mix) in a multiple linear regression analysis.

**Results:**

Between June 2014 and July 2015 the prescribing patterns of 860 GPs were analysed, among which 102 were GP trainers (12%). Over the year 363,580 patients were prescribed an antibiotic out of 3,499,248 visits for 1,299,308 patients seen over the year thus representing around 27.5% of patients. In the multivariate analyses, being a trainer resulted in a significant difference of 6.62 percentage points (IC 95%: [-8.55; -4.69]; p<0.001) in antibiotic prescriptions comparing to being a non-trainer, corresponding to a relative reduction of 23.4%.

**Conclusion:**

These findings highlight the role of GP trainers in antibiotic prescriptions. By prescribing fewer antibiotics and influencing the next generations of GPs, the human and economic burden of antibiotics could be reduced.

## Introduction

Antimicrobial resistance has become a major concern worldwide [1]. Several publications have suggested that decreasing antibiotic consumption could contribute to reducing the growth of antibiotic-resistant bacteria [2,3]. Moreover, strong evidence suggests that a substantial proportion of antibiotic prescriptions in general practice could be avoided because most infections are due to viruses [4,5] and that there is a real overuse of broad-spectrum antibiotics [6]. The issue of interventions to reduce inappropriate antibiotic prescriptions is all the more relevant given that the first case of bacterial resistance to last-resort antibiotics was reported in 2016 [7]. A Cochrane systematic review suggests that multi-faceted interventions are the best way to reduce inappropriate prescriptions [8] in combining educational interventions for General Practitioners (GPs) and practical tools easily usable in primary care [9,10].

At the end of the 1990s, outpatient antibiotic use and bacterial resistance rates in France were among the highest in Europe [11]. As a consequence, the French government launched several nationwide alert plans for antibiotics in 2002 to reduce antibiotic prescriptions and preserve antimicrobial efficiency [12]. However, the long-term effects of these plans have been disputed [13]. After a first decrease from 2002 to 2004 French antibiotic consumption remained stable since 2005 [14]. In order to provide an incentive to GPs to decrease the number of DDDs prescribed, the government developed a complementary strategy in 2012 by introducing an item for the antibiotic prescribing rate in the French National Pay for Performance System (P4P). However, the P4P scheme seem to have had non significant impact on antibiotic consumption since the number of DDD didn’t change since 2012 [14].

In contrast, no study has investigated the consequences of being a GP trainer on antibiotic prescriptions. Although GP trainers are similar to other GPs in terms of sociodemographic and medical activities [15], they may have different prescription patterns. Indeed, they must answer trainees’ questions, help them to manage their doubts and justify their behaviors, particularly in terms of prescriptions. Considering their involvement in GP education, GP trainers play a central role in improving the appropriateness of antibiotic prescriptions written by future GPs.

The objective of our analysis was to investigate whether being a GP trainer was associated with issuing fewer antibiotic prescriptions for primary care patients.

## Methods

### Data source and study design

We conducted an observational cross-sectional study using the data claims from the French National Health Insurance (NHI) for all GPs in the French department in Yvelines (78), which is a Paris suburb with a population of 1.3 million (2% of the French population). The study period ranged from June 2014 to July 2015 (one year). Information about GP trainers’ status was provided by the Department of General Practice at the University of Versailles Saint-Quentin-en-Yvelines (UVSQ) and was extracted from the French NHI data claims. All data were anonymized.

### Study sample

In 2014, 980 GPs were working in Yvelines. The exclusion criteria consisted of practicing alternative medicine (homeopathy, acupuncture, etc.) and having less than 1,000 office and home visits over the year. Information about the sociodemographic characteristics and level of activity of GPs were collected. Information about the patients was aggregated at the GP level to define the main features of the GP’s activity. Individual data for patients were not available.

### Outcome measure

The antibiotic prescribing rate was calculated as the number of patients who were prescribed at least one antibiotic by the GP over the year divided by the total of individual patients followed by the same GP during the studied period. Exhaustive information was obtained regarding the patients followed by each GP and there were no missing data.

### Variables

The main independent variable was the training status of the GPs.

We further adjusted for potential confounders selected based on the literature and their clinical relevance. These variables included GPs’ sociodemographic characteristics and location of the practice (as a proxy for physician density, which increases outpatient antibiotic consumption [16]). We also included the gender of the GP, his/her age and the level of activity, which could influence antibiotic prescription.

Adjustment of the GPs case mix was made using the proportions of visits dedicated to each type of patient as proxies. We suspected that a higher proportion of visits involving children (<16 years) or patients exempt from the medical fee (full financial coverage by the NHI) for long-term disease conditions could increase the antibiotic prescribing rate [17]. Some patients had a medical fee exemption status due to their low-income status (less than €721 per month for one person). The proportion of GPs’ visits with these financially disadvantaged patients was also determined.

### Statistical analysis

The profiles of GP trainers and non-trainers were first compared using a Chi-squared test for categorical variables and Student’s t-test for continuous variables, including the antibiotic prescribing rate.

Then, an adjusted linear regression model was used to identify factors associated with the antibiotic prescribing rate according to the training status and controlling for the main confounders as defined above.

No data were missing. All tests were two-sided, and p-values ≤0.05 were considered significant. Analyses were performed with STATA version 13.0.

### Ethics

Access to the databases is subject to prior authorization and was approved by the Independent Data Protection Administrative Authority (Commission Nationale Informatique et Libertés, CNIL). This study was approved by the Ethical Committee of the French National College of Teachers in General Practice (IRB n°00010804, Notification n°30101728). Written informed consent was not required because the data were anonymized.

## Results

### Sample characteristics

**Fig 1:**
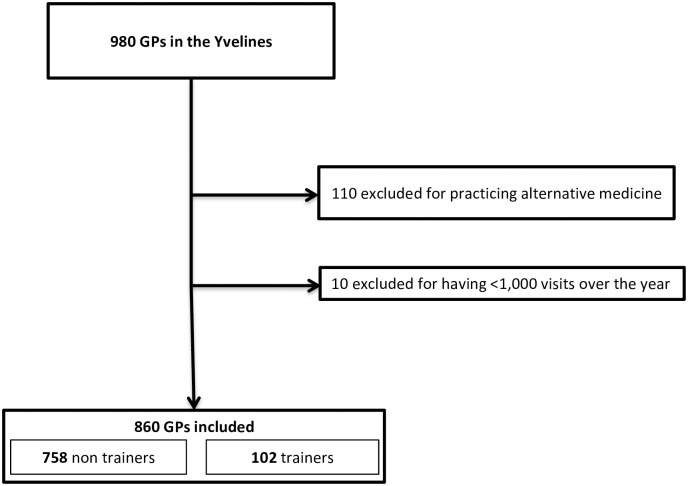
Flow chart of the study population.

Of the 980 GPs practicing in Yvelines, 120 were excluded from the statistical analyses because they practiced alternative medicine or had less than 1,000 office and home visits over the year (see Fig 1).

In total, 860 GPs were included of whom 102 were GP trainers (12%). Between June 2014 and July 2015, 3,499,248 visits occurred involving 1,299,308 individual patients. 363,580 patients had an antibiotic at least once in the year.

Our sample consisted of 64% male GPs, and 57% were over 55 years old. The GPs saw more adults than children; 21% of visits involved patients who were under 16 years old. One-fifth of home and office visits were dedicated to patients who were exempt from the medical fee: 15% for long-term disease and 6.5% for low-income status (see Table 1).

**Table 1:** Descriptive statistics of the GPs.

GPs’ characteristics	Number of GPs n = 860 (%)
***Gender***	
Male	550 (63.95)
Female	310 (36.05)
***Age***	
≥55yo	492 (57.21)
<55yo	368 (42.79)
**City size of the GP’s practice location**	
≥15,000 inhabitants	569 (66.16)
<15,000 inhabitants	291 (33.84)
**GP trainer**	
Yes	102 (11.86)
No	758 (88.14)
**GPs’ activity**	**Means (%)**
**Number of patients seen at least once over the year**	1,511
**Number of visits over the year**	4,069
Less than 3,000	264 (30.70)
Between 3,000 and 4,999	385 (44.77)
5,000 and more	211 (24.53)
**Visits’ proportions**	
dedicated to patients < 16yo	870 (21.07)
dedicated to long-term disease patients	606 (14.96)
dedicated to patients with medical fee exemption status for perceiving low incomes	308 (6.47)
**Patients to whom at least one antibiotic was prescribed**	423 (27.53)

An average of 1,500 patients attended each GP.

### Antibiotic prescribing rate

**Fig 2:**
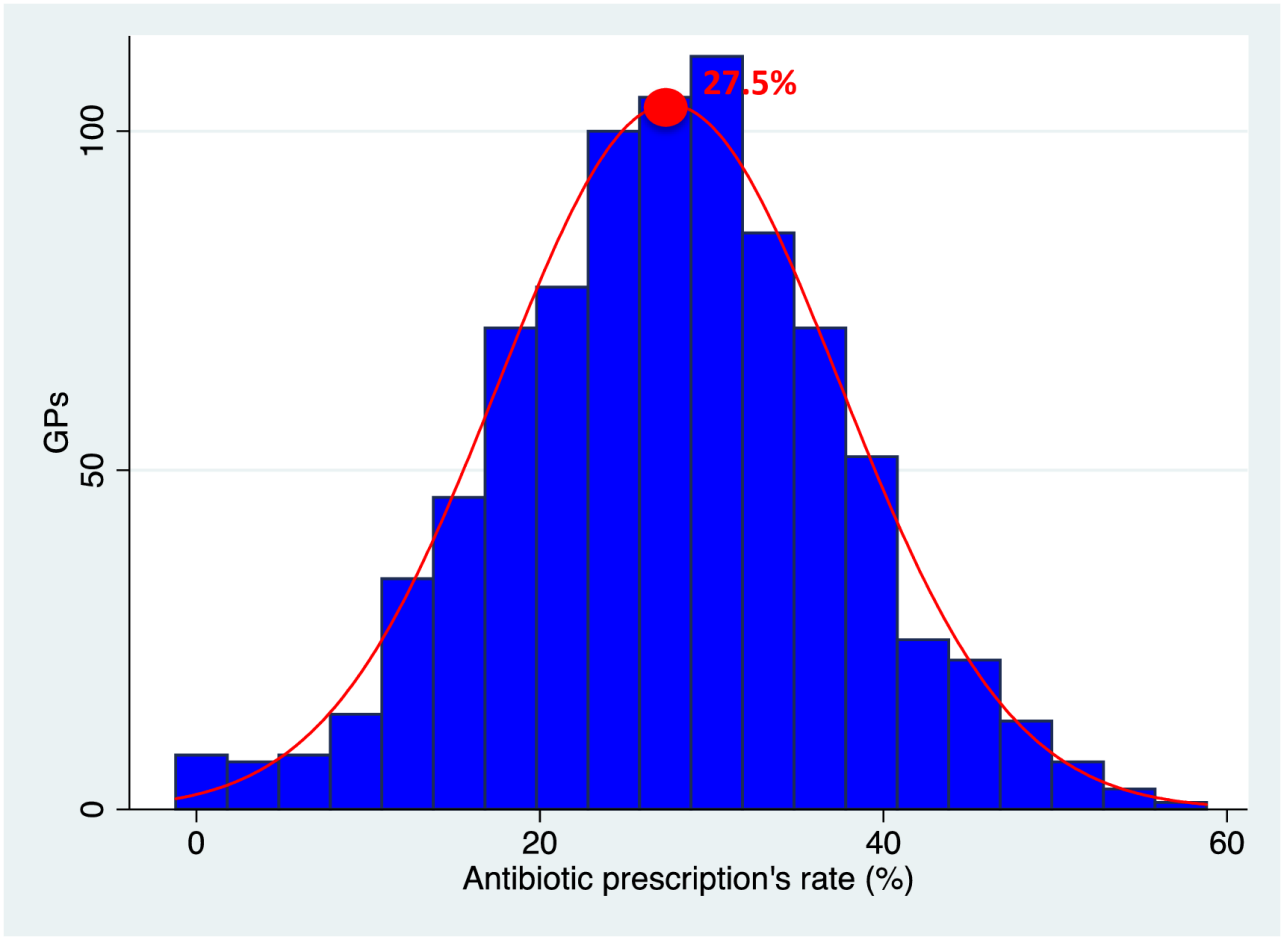
Distribution of antibiotic prescription. Lecture: The red point represents the mean antibiotic prescribing rate (27.5%).

The average antibiotic prescribing rate for the GPs was 27.53% (Q1 = 20.95%; Q3 = 34.10%) (see Fig 2).

Regarding the results for trainers and non-trainers, the antibiotic prescribing rate was significantly lower among trainers: **21.86%** compared to **28.29%** (p<0.001) (see Table 2).

**Table 2:** Bivariate analysis.

	GP trainers (n = 102)	Other GPs (n = 758)	p value
***Gender***			0.54
Male	66.67%	63.59%	
Female	33.33%	36.41%	
***Age***			0.62
≥ 55 yo	54.90%	57.52%	
< 55 yo	45.10%	42.48%	
***City size of the GP’s practice location***			0.74
≥ 15,000 inhabitants	67.65%	65.96%	
< 15,000 inhabitants	32.35%	34.04%	
**GP’s level of activity over the year: mean**			
Number of patients	1,462	1,517	0.5
Number of visits	3,884	4,094	0.3
Proportion of attendances by patients < 16yo	21.83%	20.97%	0.27
Proportion of attendances by patients ≥ 16yo	78.16%	79.03%	0.26
Proportion of attendances by patients with long-term disease	14.92%	14.97%	0.93
Proportion of attendances by patients with medical fee exemption status for perceiving low incomes	5.35%	6.62%	0.07
**Proportion of patients to whom at least one antibiotic was prescribed: mean**	**21.86%**	**28.29%**	**<0.001**

### Factors associated with antibiotic prescriptions

Table 3 presents the results of the regression. In the unadjusted model, GP trainers’ antibiotic prescription rates are significantly lower compared to non-trainers’ by 6.43 percentage points (CI 95%: [-8.43; -4.42]; p<0.001).

**Table 3:** Results of the multivariate regression analyses.

	Non Adjusted (R^2^ 0.04)			Ajusted (R^2^ 0.14)		
	*Coef*.	*CI 95%*	*p value*	*Coef*.	*CI 95%*	*p value*
**GP Trainer**						
Yes	-6.43	[-8.43; -4.42]	<0.001	-6.62	[-8.55; -4.69]	<0.001
No	Ref.			Ref.		
**Gender**						
Male	-	-	-	0.34	[-1.05; 1.73]	0.63
Female	-	-	-	Ref.		
**Age**						
≥ 55yo	-	-	-	3.67	[2.31; 5.02]	<0.001
< 55yo	-	-	-	Ref.		
**Location of the GP’s practice**						
≥ 15,000 inhabitants	-	-	-	0.36	[-1.01; 1.75]	0.61
< 15,000 inhabitants	-	-	-	Ref.		
**Level of activity (Number of visits)**						
<3,000	-	-	-	-3.81	[-5.28; -2.35]	<0.001
3,000–4,999	-	-	-	Ref.		
≥5,000	-	-	-	3.52	[1.86; 5.17]	<0.001
***Proportion of attendances by patients***						
< 16yo	-	-	-	0.13	[0.02; 0.25]	0.02
with long-term disease	-	-	-	0.09	[-0.07; 0.23]	0.291
with medical fee exemption status for perceiving low incomes	-	-	-	-0.19	[-0.30; -0.08]	0.001

Reading grid: GP for General Practitioner, CI for Confidence Interval, yo for years old.

The adjusted model showed a significant average of 6.62 percentage points lower for the GP trainers’ antibiotic prescribing rate compared to non-trainers. (23.4% in relative terms)

Compared to the univariate analysis, the multivariate analysis showed a stronger impact of being a GP trainer versus non-trainer on antibiotic prescribing: resulting in a significant difference of 6.62 percentage points (CI 95%: [-8.55; -4.69]; p<0.001). In relative terms, this effect represents a difference of 23.4% between the two groups.

Being 55 years old or older increased the antibiotic prescribing rate by an average of 3.67 percentage points (CI 95%: [2.31; 5.02]; p<0.001) with all other factors being equal. Regarding the level of activity, as expected GPs with a higher workload prescribed more often antibiotics to their patients (+3.52, CI 95%: [1.86; 5.17]). In contrast, for GPs with fewer than 3,000 annual visits, the antibiotic prescribing rate was reduced by 3.81 percentage points on average (reference: 3,000 to 4,999 annual visits).

An increase in the annual number of attendances by patients <16 years old of 10 percentage points was associated with an average increase of 1.3 percentage points in the antibiotic prescribing rate (CI 95% [0.2; 2.5]; p = 0.02). The annual number of attendances by patients with an exemption for medical fees (individuals with low income) was statistically significant (p = 0.001): for every 10 percentage points increase in the number of visits dedicated to these types of patients, the antibiotic prescribing rate increased by 1.9 percentage points on average CI 95% [-3.0; -0.8].

## Discussion

### Summary of results and comparisons to the literature

After adjusting for several confounders, we have shown that GP trainers’ antibiotic prescription rates were significantly lower compared to non-trainers’ by 6.62 percentage points (CI 95%: [-8.55; -4.69]; p<0.001), thus corresponding to a relative difference of 23.4%.

To the best of our knowledge, this is the first study to show a significantly lower antibiotic prescribing rate for GP trainers than non-trainers.

Our results are consistent with previous studies that reported that GP trainers tended to have better clinical performances based on P4P indicators, such as diabetes follow-up, seasonal flu vaccination and mammograms [15].

Multifaceted interventions and educational programs are efficient and have a positive effect on GP prescriptions [18,19]. They involve multiple health care providers, considerable resources and diverse training materials and methods. Educational methods help to address the deep reasons for unnecessary antibiotic prescription, and improve reflection on practice based experience in order to understand when, why and how GPs can improve their practice. Reflective practice [20,21] might be a key reason explaining why GP trainers prescribe fewer antibiotics, thanks to initial medical education and continuing medical education. Moreover tutoring, teaching, and being exposed to students and their questions, may also be important factors explaining our results.

The antibiotic prescribing rate increased with the number of patient visits. Indeed, in a fee-for-services payment scheme, there is a trade-off between the number and duration of consultations: GPs who conduct more examinations per day spend less time with patients [22], which makes it more difficult for the GP to argue when the patient requests antibiotics [23].

Our study could not confirm a previous finding that older patients are more likely to be prescribed antibiotics [6,24].

### Strengths and limitations

Our sample included all GPs with regular practice activity in Yvelines (almost 90% of all GPs), and all GP trainers were included. The large number of patients (almost 1.3 million) suggests good coverage of the population of Yvelines (90%). The main characteristics of the inhabitants of Yvelines were similar to the French population, including approximately 51% of women vs 52% in France overall. The age distribution of the inhabitants was similar, with approximately 38% under 30 years old in the Yvelines versus 36% in France, 54% from 30 to 74 years old vs 55% and 7% vs 9% 75 years old and over [25]. Finally, the number of GPs per 100,000 inhabitants of Yvelines was lower than the French GP density: 105 vs 132 [26].

The proportion of GP trainers was similar to the national level: 12% vs 13% for Yvelines and France, respectively. Moreover, GP trainers were comparable to non-trainers in terms of sociodemographic characteristics and case-mix, as shown in other studies [15,27]. Although our conclusions may not be generalizable to all French GP trainers because we included GP trainers from only one General Practice Department, we had no missing data.

We proposed to assess the prescriptions of the GPs by using the antibiotic prescribing rate in order to have a better reflect of the habits of GPs and answer our question [28]. This outcome is often reported in literature [29]. The standard measurement of antibiotic consumption with DDD was not as relevant for our study.

Our study has several limitations because some variables that could have an effect on antibiotic prescriptions were not collected. First, we did not have any information about the type of disease that was treated or the list of antibiotics used. A systematic review about factors associated with antibiotic prescriptions for respiratory tract infections (RTIs) found that symptoms and diagnoses played a role in antibiotic prescribing rates [30]. Indeed, RTIs or urinary tracts infections (UTIs) are often responsible for some unjustified or inappropriate prescriptions [31,32]. The only clinical information available regarding the disease was whether the patients had a long-term disease, but we did not have access to the justification of the consultation for antibiotics.

Our study provides evidence that GP trainer status affects prescription behavior. But other variables that were not adjusted for could be confounding factors that could explain the high and strongly significant coefficient for the training status. Residual confusion remains possible in GP trainers’ characteristics such as practicing in groups, having additional diplomas and examining fewer patients [27]. Factors such as reading medical journals, using rapid tests or having discussions with patients about their prescriptions also have a positive effect on following guidelines for antibiotic prescriptions [33]. Because of the cross sectional nature of the data, it was not possible to separate the pure effect of being a GP trainer from the selection effect (GPs could already prescribe fewer antibiotics before becoming trainers).

### Implications for research and practice

In the Yvelines for 100 patients followed over one year, a GP trainer prescribes an antibiotic to 6 patients fewer than a non-trainer. If we extrapolate our results, considering that a GP examines approximately 1,500 patients per year: 90 patients would not be treated by an antibiotic in one year. Extrapolated to all French GP trainers (8,550) [34], 769,500 patients in total would not be treated by antibiotics over one year.

By prescribing fewer antibiotics we suppose that GP trainers could influence the next generations of GPs [35,36] and that they might participate in reducing the human and economic burdens of antibiotics [37] by many other ways. Thus, it is important to focus on the prescription patterns of GP trainers, even if the design of the study does not allow our results to be used to promote additional training or recruitment of more GPs based on this argument alone. The effect on the next generations of GPs has indeed not yet been proven. It would be also very interesting to explore the role of patient’s education during visits to observe if there is a change in patients’ will of antibiotics.

Further studies are needed to assess the differences between GP trainer and non-trainer in terms of prescription patterns.

A follow-up or retrospective study to assess the antibiotic prescribing rates of GPs from the same generation before and after becoming a trainer would be informative. This type of study could examine the changes (or lack of changes) in the behavior of GPs concerning antibiotic prescriptions. It would also be possible to study a larger population of GPs from other French departments to define the factors or mechanisms that explain the lower rates of antibiotic prescriptions, such as continuing medical education, reading medical papers, going to conferences, tutoring, training students, having a reflective attitude during consultations, accepting or not accepting medical sales representatives, and asking the patients if they want an antibiotic.

In addition to this quantitative study, a qualitative study would increase our understanding of the perceptions patterns of GPs upon becoming trainers. We could enhance the work by exploring how patients feel about antibiotics if their GP is a trainer or not. Further studies could also focus on the trainee’s prescriptions and how they justify these prescriptions based on their trainer’s behavior during their internship.

## Conclusion

It is possible to become more vigilant about antibiotic prescriptions and decrease the burden of antibiotic resistance. A post-antibiotic era [1] is an actual threat that should be taken very seriously. It has become essential to do everything possible to reduce the rate of antibiotic prescription. For the first time, we have shown that being a GP trainer was significantly associated with a lower antibiotic prescribing rate for any diagnosis. Trainers inducting appropriate behavior for students and for patients could be one answer to the problem of antibiotic prescription and consumption that is still to explore with further studies.

## Supporting information

S1 Fig The French context. (TIFF)

## Abbreviations

CI: Confidence Interval
DDD: Defined Daily Dose
GP: General Practitioner
NHI: French National Health Insurance
P4P: Pay for Performance
RTI: Respiratory Tract Infection
UVSQ: University of Versailles Saint-Quentin-en-Yvelines
