# Supplementary figures and images for: General Practitioner trainers prescribe fewer antibiotics in primary care: Evidence from France
Source: PLoS One. 2018 Jan 25;13(1):e0190522. doi: 10.1371/journal.pone.0190522 (PMC5784911; doi:10.1371/journal.pone.0190522)

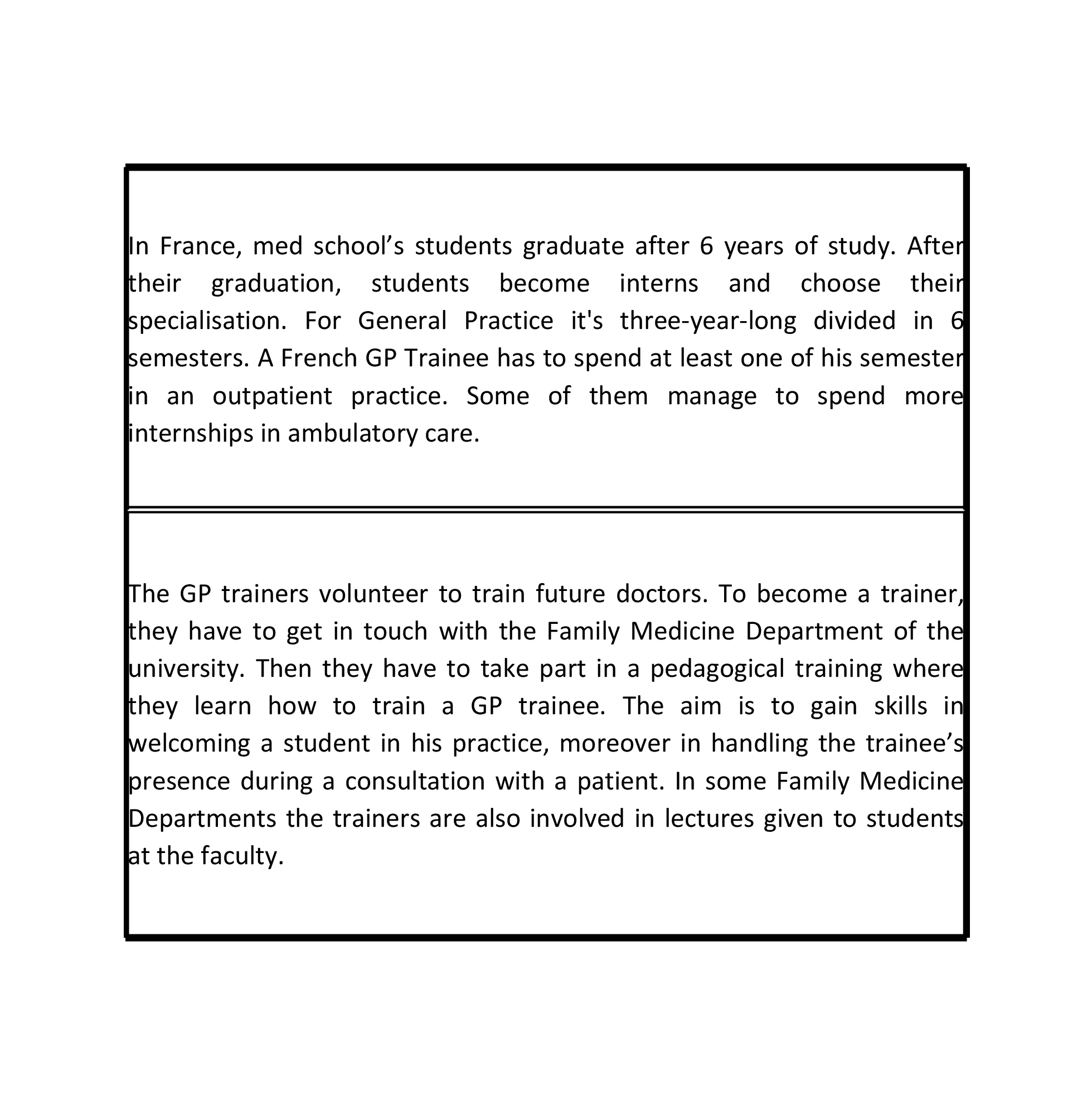

Supplement: S1 Fig — (TIFF) [file pone.0190522.s001.tiff]
